# Data saves lives: optimising routinely collected clinical data for rare disease research

**DOI:** 10.1186/s13023-023-02912-1

**Published:** 2023-09-11

**Authors:** Ameenat Lola Solebo, Pirro Hysi, Lisanne Andra Horvat-Gitsels, Jugnoo Sangeeta Rahi

**Affiliations:** 1 Population, Policy and Practice Research and Teaching Department, Great Ormond Street Institute of Child Health, University College London, 30 Guilford Street, London, WC1N 1EH UK; 2 Ulverscroft Vision Research Group, Great Ormond Street Institute of Child Health, University College London, London, UK; 3 Great Ormond Street Hospital for Children, NHS Foundation Trust, London, UK; 4 Section of Ophthalmology, School of Life Course Sciences, King’s College London, London, UK; 5 Department of Twin Research and Genetic Epidemiology, School of Life Course Sciences, King’s College London, London, UK; 6 Institute of Ophthalmology, University College London and NIHR Moorfields Biomedical Research Centre London, London, UK

**Keywords:** Electronic health records, Information management, Rare disease, Translational research, Biomedical, Epidemiology

## Abstract

Necessity driven organisational change in the post-pandemic landscape has seen health care providers adopting innovations to manage and process health data. These include the use of ‘real-world’ datasets of routinely collected clinical information, enabling data-driven delivery. Rare disease risks being ‘left-behind’ unless our clinical and research communities engage with the challenges and opportunities afforded by the burgeoning field of health data informatics. We address the challenges to the meaningful use and reuse of rare disease data, and, through a series of recommendations around workforce education, harmonisation of taxonomy, and ensuring an inclusive health data environment, we highlight the role that those who manage rare disease must play in addressing them.

Despite the significant direct and indirect negative impact of the COVID-19 pandemic on global health, the disruption to health care services has, in some areas, resulted in opportunities for the advancement of patient care [1]. Necessity driven organisational change has seen health care providers adopting innovations to manage and process health data [2, 3]. A next step in addressing the complex challenges of longer-term restoration of quality care will be the operationalisation of ‘real-world’ datasets of routinely collected clinical information. This will enable data-driven delivery of care by supporting agile pragmatic or adaptive studies anchored in ‘real-life’ data [4]. These advances are only possible with stakeholder engagement, particularly of those clinical teams who generate these data. Rare disease—collectively affecting 3.5–6% of the population, an estimated 263–446 million persons[5]—risks being ‘left-behind’ unless our clinical and research communities engage with the challenges and opportunities afforded by the burgeoning field of health data informatics. Meaningful use of health data is all the more important in areas where those health data are particularly scarce, and the individual rarity of these uncommon disorders magnifies the adverse impact to the evidence base of ‘data wastage’ through failure to take advantage of appropriate design and implementation of health informatic platforms and applications.

## Not all health informatic systems are created equal

**Fig. 1:**
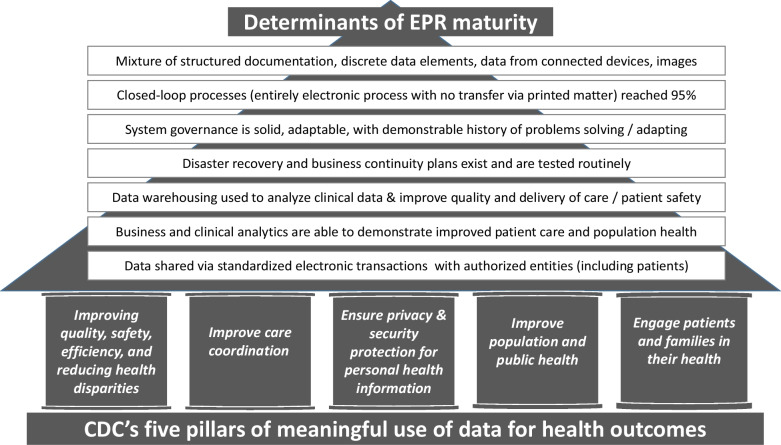
The requisites of the maturity of electronic health records are determined by the priorities for patient and population health outcomes [6]

Electronic health records—also termed electronic patient or electronic medical records in different clinical contexts—vary in quality and usability. Maturity is a key metric of EHR robustness which speaks to the stability, responsiveness, interoperability and usability of the system, as well as the measurable benefit to patient care and the wider population. This measurable positive impact lies at the heart of the importance of EHR maturity, with ideal system being one which enables meaningful use of data for the delivery of high quality, equitable patient-centred care, consistent with national (eg, the US Centre for Disease Control, Fig. 1) [6] and supranational (eg, the United Nations) [7] definitions of such care.

Mature EHR systems support the operationalization of data [8]. The essential requisites for a mature EHR are laid out in the Healthcare Information and Management Systems Society’s inpatient and outpatient Electronic Medical Record Adoption Model (EMRAM and (O)EMRAM, Fig. 1). It is important that clinicians understand these requisites and are familiar with the issue of maturity when called on to engage with the choice and adoption of EHR systems within their practice. However, clinicians should also be aware that the maturity of these systems is irrelevant to the meaningful use of health data without an infrastructure for system implementation. The success of this infrastructure is dependent on their teams. We address the challenges to the meaningful use and reuse of rare disease data, and, through a series of recommendations, we highlight the role that those who manage rare disease must play in addressing them.

## Education: data literacy skills for clinical and non-clinical health care staff

The digitisation of data within a health care setting is “adaptive change of the highest order” [9], irrevocably changing the nature of work and those who do the work [10]. Data management skills are a necessary workforce prerequisite for the successful implementation of an electronic health records system.

Within a health care system, data flows in a cyclical fashion, from generation (by patients or clinicians), to storage, processing, analysis and the use of that analysis to impact care and subsequent generation of data. Weak links in this data cycle limit the effectiveness of analysis and resultant application of data. This health data cycle is particularly vulnerable in rare disease. The evidence base which supports improvements in rare disease care and services is reliant on studies with small population sizes, where the scarcity of generated data makes efficient use of that data critically important. Rare disease care is also reliant on multi-centre collaborations, where efficient processing (harmonisation and integration) and analysis is dependent on the quality of the generated data.

Increasingly, medical schools and nurse training courses include modules on data management, but this is not routinely offered to allied health professionals or non-medical staff, all of whom generate and use data within health care settings. New staff joining a care facility typically have inductive training in the relevant EHR system, but not on the principles of the health data cycle or the importance of understanding the structure of data. Cyclically updated training breeds confidence with EHR interfaces and usability [11], and supports staff in driving ongoing optimisation of the EHR interface, with measurable, significant benefit of staff experience [12]. A data-literate clinical workforce also understands that the ‘findable, accessible, interoperable and reusable’ (FAIR) principles and standards apply to all kinds of data [6, 8, 9, 12]. These data types include lists of eligibility criteria for rare disease registries, or a database of local allied care centres with the capacity to co-manage rare and complex disease.

For large, complex and rapidly evolving datasets, the burden of implementing FAIR standards can be considerable. There will be an increasing role of artificial intelligence in making data findable (eg, through searches improved by natural language processing approaches), accessible (eg through adaptable interfaces for those with disabilities), interoperable (eg through algorithmic dataset harmonisation) and reusable (eg by automating data cleaning or transformation for different purposes). This increasing role will still be reliant on stakeholders. Clinicians and researchers need to come together to consider what ‘FAIR’ looks like, and thus to define the value and utility of data and metadata.

*Recommendation*: All staff who interact with health data should receive ongoing training in the principles of the health data cycle, and the necessity of ensuring that data are findable, accessible, interoperable, and reusable (FAIR).

## Capacity: increase the critical mass of subject matter experts trained in health informatics

Data literacy enables an individual to begin to understand what it takes to ‘ask good questions’ of the data stored within their EHRs [13]. Patient-facing clinicians often originate these ‘good questions’ but answering them in rare disease using routinely collected clinical data requires datasets gathered across multiple centres which are sufficiently granular as to allow description and evaluation of complex phenotypes. This typically requires additional analytic and programming skills. EHRs contain highly structured data comprising quantitative or qualitative variables such as age, body mass index, drug names, but may also contain ‘dirty’ or unstructured data, such as free text entries. Free text data within EHRs can hold valuable information on patient experience, disease severity, reported adverse events, or details on concordance with prescribed medication. However, without subject matter expertise, these data are a challenge to transform into a product that can be queried and analysed [14, 15]. The analyses of such data require individuals who understand both the clinical question and context and the capabilities of analytical platforms and programming languages such as the open-source R, SPARQL and Python languages [16]. User-friendly interfaces for these programming languages are available, allowing for intuitive use of these tools to analyse or visualise data, without the need for deep coding knowledge. Wide adoption of these skills may also improve the working experience, health, and wellbeing of the staff members themselves. The great promise of the digitisation of health care is the eventual ‘gift of time’ for healthcare workers.[12] In some settings, eg those which lack the resource of data science staff to support the informed use of information, digitisation is more likely to contribute to physician stress and burnout [17]. Rather than the redirection of attention from the patient which is often noted by clinicians following their hospital’s adoption of HER [18], implementation of intelligent EHR systems may free clinical staff to spend more time interacting with their patients. This will be particularly important during public health crises [19]. The ‘artificial intelligence’ of the system will be dependent on the ‘good questions’ it has answered, and how it has answered them, and this depends in turn on the involvement of the subject matter expertise of hospital staff.

*Recommendation*: The creation and expansion of a rare disease analyst workforce, with data skills present even in staff who lack the terms ‘coding’ or ‘analysis’ in their work title, and subject matter experts with health informatics experience, is urgently needed, and should be a priority across all health care settings.

## Collaboration: maintaining metadata

Health service delivery for rare disease involves care across and within different tiers, from primary care to super-specialised tertiary team structures, to links with national or international disease registries, and links with external regulatory authorities (e.g., tissue and transplant authorities). Communication across these settings is negatively impacted by the siloed approach to data collection which characterises most health care settings [15]. Whilst the structure of the data generated within the EHR of individual care settings may meet the metadata-related requirements necessary to establish FAIR use, this is not always true of the other datasets, particularly those datasets lacking metadata, ie descriptive information on data elements, dataset structure, location of data storage, and provenance [20]. An illustrative example is absence of uniform adoption of the Digital Imaging and Communications in Medicine, or DICOM metadata standards [15, 16]. Imaging is particularly important for objective capture of phenotype in rare diseases, which tend to be characterised by heterogeneity and complexity. The DICOM metadata standards ensure documentation of the descriptive data (image type, mode of acquisition, image machine settings) needed to ensure that the images are accessible to and usable by other clinicians and researchers [15, 16]. Meta-data capture and data cataloguing thus reduce the risk of ‘health data entrapment’, where critical data are less accessible or interoperable [21, 22].

*Recommendation*: Clinical and clinical research teams should ensure the creation of detailed metadata (such as modes of data acquisition, authorship details, timestamps) for rare disease study datasets, registries or other data item, to ensure dataset re-use.

## Standardization: medical terminology, coding and cataloguing

Prompt identification of specific populations of patients allows for redirection of care, audit of clinical outcomes, and can support recruitment to time-sensitive research. These forms of data utilisation are reliant on a high degree of clinical terminology harmonization among EHR users. During the pandemic, multi-centre collaborative networks of researchers worked together to develop COVID-related projects, with subject matter experts (clinicians) and health informaticians co-developing search algorithms within the EHR to generate lists of eligible individuals for inclusion within the studies. Clinical data have also been used for pragmatic and adaptive randomised controlled trials [5]. Terminology on key clinical elements such as clinical condition is based on the World Health Organization’s International Classification of Diseases (ICD) taxonomy.

**Fig. 2:**
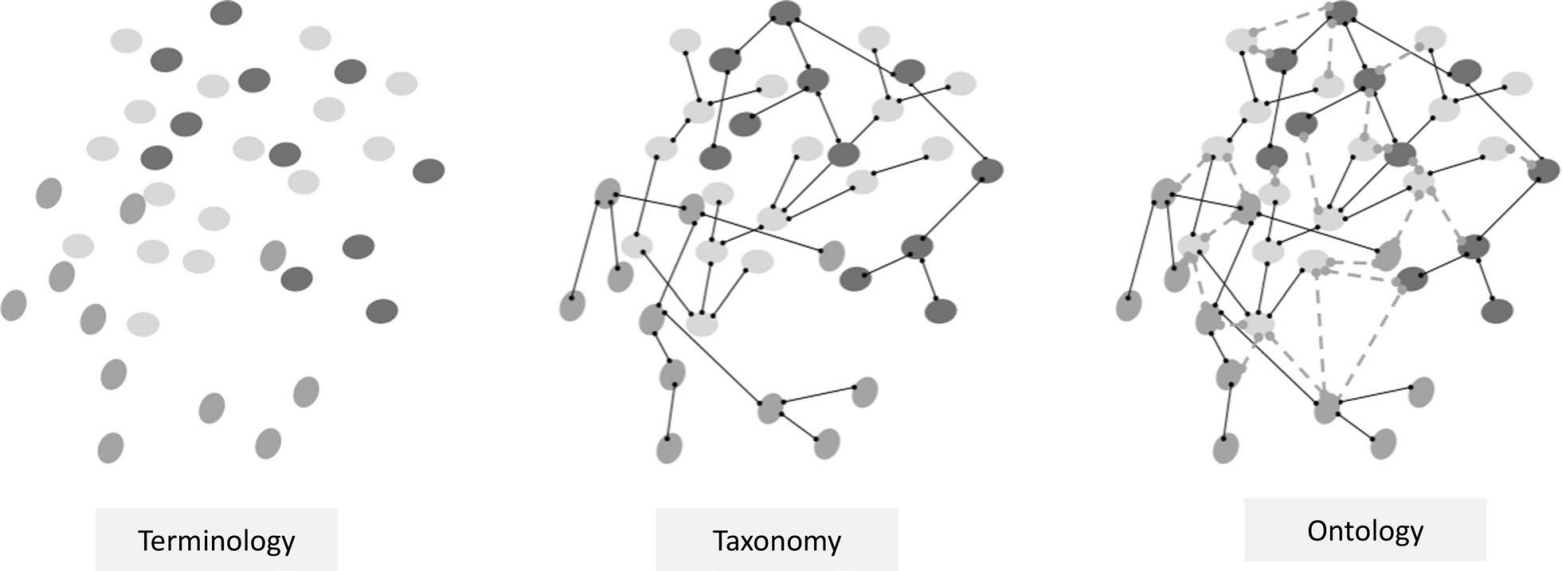
Terminology, taxonomy and ontology

The words terminology, taxonomy and ontology are often used interchangeably but have different meanings (Fig. 2). Ontologies provide context for data, by ensuring representation of the relationships between concepts and entities. This supports the complexity needed to integrate and standardise data on related concepts from different sources, and supports the logical reasoning needed to make inferences, conclusions or decisions about data. The Systematized Nomenclature of Medicine Clinical Terms (SNOMED CT) is now, internationally, the leading clinical and healthcare ontology [23]. For example, it supports the multiple synonyms typically associated with distinct concepts. Examples of this include pneumonia, which has over 1000 synonyms including ‘bronchitis’ or chest infection’, or concepts such as ‘body weight’, which can be considered a clinical finding, a disorder in individuals with clinical obesity, and an entity seen in context with medications prescribed by body weight. This growing granularity of the EHR environment allows richer capture of concepts such as findings, interventions, pharmaceutical or biologic products, geographical location or social context. However, it can become an obstacle to data utilisation without the involvement of subject matter experts to identify and report associative relationships. Progress requires clinicians / subject matter experts to collaborate to create consensus-based libraries of SNOMED CT-defined conditions, which can then be shared or accessed more widely for clinical or analytic purposes [5, 24].

The standardisation of nomenclature is particularly important for the future ‘intelligent’ (ie, data-driven) use of EHR data. The healthcare workforce has been under considerable strain during the COVID-19 pandemic, and now faces the burden of rebuilding non-COVID related care to avoid reversing the earlier gains made in rare disease. Artificial intelligence-based predictive analytics, embedded into EHR systems, and able to personalise treatment by modelling prognosis and treatment response, may be able to release clinician time [12]. The development and maturity of such AI assistance will, to a great extent, be dependent on the development of large, carefully coded and ontologically annotated datasets. Without these datasets, we will lack the domain specific and contextual understanding needed to train AI efficiently. The annotation of these datasets is time-consuming, and resource intensive with regards to the hours needed from clinical subject matter experts. Future ‘hybrid’ AI approaches will enable the use of unannotated or unstructured data for the training of assistive health care AI, providing the adaptability necessary to meet novel or unforeseen challenges such as the emergence of new diseases or significant shifts in health care delivery structures. Until then, the harnessing of supportive AI health tech will depend on datasets which are annotated with explicit definitions of the meanings and relationships contained within them, and those annotations must be anchored within subject matter expertise.

*Recommendations*: Health care professionals who manage rare disease must take up leadership roles in data science and establish collaborative networks to develop consensus led data libraries.

## Inclusivity: equitable patient access to health care information

International differences in implementation of EHR systems are well-documented, with adoption of EHRs being much lower on average in the lower-middle (35% of whom have adopted EHRs) and low-income countries (15%), compared with > 50% in upper-middle- and high-income countries [25]. However, data poverty, where families are unable to access online services due to educational, financial or geographical limitations is not necessarily predictable by national GDP (gross domestic product). One in six adults in the majority of all Organization for Economic Co-operation and Development (OECD) countries are at the lowest level of literacy (Level 1, basic vocabulary only, unable to make low-level inferences, and unable to make matches between the text, either digital or printed, and information) [26]. The importance of the individual’s right of access to a computable version of their medical record is widely recognised [7, 27], but although EHR implementation has been associated with improved health outcomes, that association is weakest for those in their country’s lowest socioeconomic strata [28], and there is under-ascertainment of families with low data literacy [29, 30]. Additionally, data governance will be an important consideration when communicating the importance of data use to communities, especially in the context of disengagement from health care professionals, issues around trust, or health misinformation. Transparency around ownership, use and protection of data will be of great importance, particularly where patients are being asked to share data they have generated and which, as with all the data generated about them as patients or service users, they own.

Although EHRs bring many advantages for rare disease patients, empowering them as they receive care from multi-disciplinary teams across multiple centres, or as they transition to different models of complex care, care must be taken to avoid exacerbating existing health and disease outcome disparities. Those who design and implement EHR systems must address how they can maintain patient trust, support wide and equitable accessibility for patients to their health care data [31, 32], and avoid widening the existing disparities in health care access and health outcomes [33].

*Recommendation*: The system-level, rather than patient-level drivers behind inequitable EHR impact must be considered during EHR implementation, and clinical teams must also consider whose data are, and whose data are not being collected within the EHR.

## Conclusion

Mature EHR systems are those which address the optimisation of care processes and patient health outcomes, through prediction and prevention of unwanted patient experience or health outcomes [25]. The success of these systems is dependent on their implementation, and the delivery of such systems are critical national and international goals [7, 20, 25]. Future responses to national health care emergencies will be driven by data [25, 34], and therefore will require robust, unbiased transparent data collection and management methods in place. Without this, data-based diagnostic and prediction models, especially those using artificial intelligence approaches, will be at high risk of amplifying bias, with resultant over-optimistic estimates of accuracy and performance [35]. Beyond the pandemic, well designed and implemented EHR will enable alignment of clinical data with a broad range of national and international rare disease health policies. The success of such policies aimed at ‘building back better’ will rests on the strength of our ‘analyst workforce’, our terminology harmonisation, our metadata, and the accessibility of patients and families to their health data.

## Data Availability

All available data and materials are presented in the article.

## Abbreviations

CDC: Centre for disease control
EHR: Electronic health record
FAIR: Findable, accessible, interoperable, reusable
GDP: Gross domestic product
ICD: International classification of diseases
UN: United Nations
WHO: World Health Organization
